# Celiac Disease Prevalence Is Increased in Primary Sjögren’s Syndrome and Diffuse Systemic Sclerosis: Lessons from a Large Multi-Center Study

**DOI:** 10.3390/jcm8040540

**Published:** 2019-04-19

**Authors:** Elena Bartoloni, Onelia Bistoni, Alessia Alunno, Lorenzo Cavagna, Linda Nalotto, Chiara Baldini, Roberta Priori, Colomba Fischetti, Micaela Fredi, Luca Quartuccio, Francesco Carubbi, Carlomaurizio Montecucco, Andrea Doria, Marta Mosca, Guido Valesini, Franco Franceschini, Salvatore De Vita, Roberto Giacomelli, Giulia Mirabelli, Vittorio Bini, Armando Gabrielli, Carlo Catassi, Roberto Gerli

**Affiliations:** 1 Rheumatology Unit, Department of Medicine, University of Perugia, 06128 Perugia, Italy ; elena.bartolonibocci@unipg.it (E.B.); o.bistoni@libero.it (O.B.); alessia.alunno82@gmail.com (A.A.); mirabelligiulia83@gmail.com (G.M.); 2 Department of Rheumatology, University and IRCCS Foundation Policlinico S. Matteo, 27100 Pavia, Italy; lorenzo.cavagna@unipv.it (L.C.); montecucco@smatteo.pv.it (C.M.); 3 Division of Rheumatology, Department of Medicine, University of Padua, 35128 Padova, Italy; nalinda@libero.it (L.N.); adoria@unipd.it (A.D.); 4 Rheumatology Unit, Department of Clinical and Experimental Medicine, University of Pisa, 56126 Pisa, Italy; chiarabaldini74@gmail.com (C.B.); marta.mosca@med.unipi.it (M.M.); 5 Department of Internal Medicine and Medical Specialties, Rheumatology, Sapienza University of Rome, 00185 Rome, Italy; roberta.priori63@gmail.com (R.P.); guido.valesini@uniroma1.it (G.V.); 6 Clinical Medicine, Department of Internal Medicine, Ospedali Riuniti University Hospital, 60030 Ancona, Italy; colomba.univpm@gmail.com (C.F.); a.gabrielli@staff.univpm.it (A.G.); 7 Department of Rheumatology and Clinical Immunology, ASST Spedali Civili, 25133 Brescia, Italy; fredi.micaela@gmail.com (M.F.); franceschini@bresciareumatologia.it (F.F.); 8 Department of Medical and Biological Sciences, Rheumatology Clinic, Santa Maria della Misericordia University Hospital, 33100 Udine, Italy; luca.quartuccio@uniud.it (L.Q.); devita.salvatore@aoud.sanita.fvg.it (S.D.V.); 9 Rheumatology Unit, Department of Biotechnological and Applied Clinical Sciences, University of L’Aquila, 67100 L’Aquila, Italy; francescocarubbi@libero.it (F.C.); roberto.giacomelli@cc.univaq.it (R.G.); 10 Internal Medicine, Endocrine and Metabolic Science Section, University of Perugia, 06128 Perugia, Italy; vittorio.bini@unipg.it; 11 Department of Pediatrics, Marche Polytechnic University, 60121 Ancona, Italy; c.catassi@univpm.it

**Keywords:** celiac disease, autoimmune rheumatic diseases, systemic lupus erythematosus, Sjögren’s syndrome, systemic sclerosis

## Abstract

Association of celiac disease (CD) with systemic autoimmune diseases (ADs) remains controversial. Awareness of CD in these patients is important to prevent complications, including lymphoproliferative disorders. We evaluated previously diagnosed CD prevalence in systemic lupus erythematosus (SLE), primary Sjögren’s syndrome (pSS) and systemic sclerosis (SSc) patients in comparison to 14,298 matched controls. All patients were screened for subclinical CD. Data from 1458 unselected consecutive SLE (580), pSS (354) and SSc (524) patients were collected. Previously biopsy-proven CD diagnosis and both CD- and AD-specific features were registered. All patients without previous CD were tested for IgA transglutaminase (TG). Anti-endomysium were tested in positive/borderline IgA TG. Duodenal biopsy was performed in IgA TG/endomysium+ to confirm CD. CD prevalence in AD was compared to that observed in 14,298 unselected sex- and age-matched adults who acted as controls. CD was more prevalent in pSS vs controls (6.78% vs 0.64%, *p* < 0.0001). A trend towards higher prevalence was observed in SLE (1.38%, *p* = 0.058) and SSc (1.34%, *p* = 0.096). Higher CD prevalence was observed in diffuse cutaneous SSc (4.5%, *p* ≤ 0.002 vs controls). Subclinical CD was found in two SLE patients and one pSS patient. CD diagnosis usually preceded that of AD. Primary SS and SSc–CD patients were younger at AD diagnosis in comparison to non-celiac patients. Autoimmune thyroiditis was associated with pSS and CD. CD prevalence is clearly increased in pSS and diffuse SSc in comparison to the general population. The association of CD with diffuse but not limited SSc may suggest different immunopathogenic mechanisms characterizing the two subsets. CD screening may be considered in pSS and diffuse SSc in young patients, particularly at the time of diagnosis.

## 1. Introduction

In recent years, advances in the understanding of the etiopathogenesis of celiac disease (CD), one of the most common gastrointestinal disorders affecting about 1% of the European population, clearly highlighted the evidence that CD should not be regarded as a mere gastrointestinal malabsorptive disorder [1,2]. In fact, the strong histocompatibility leucocyte antigen (HLA)-associated genetic background and the presentation of gluten deamidated antigen to CD4^+^ naïve T-cells by HLA-DQ2 and -DQ8 represent a clear demonstration of an autoimmune pathogenic mechanism [2]. Moreover, the presence of an environmental factor as a disease trigger, the evidence of an adaptive immune-mediated response with specific auto-antibody (Ab) production and the systemic nature of the disease (in which gastrointestinal manifestations are important but not exclusive) are adjunctive factors [3]. In this setting, CD shares many similarities with systemic and organ-specific autoimmune diseases (ADs) and several ADs are associated with CD. About 30% of patients with CD have concomitant AD in comparison to the low frequency, ranging from 3% to 9%, reported in the general population. In addition, increased prevalence of systemic or organ-specific ADs have been also described in family members of CD patients, thereby further supporting the systemic autoimmune background of the disease [4]. In particular, CD has been found in 4–11% of patients with type 1 diabetes mellitus and in 2–7% of subjects with autoimmune thyroiditis and multiple concomitant factors (including a shared genetic predisposition, immunological mechanisms and/or exposure to a common triggering event) may explain such coexistence [4]. In this setting, the recommendation suggested by the National Institute of Clinical Excellence for routine CD screening in patients with type 1 diabetes mellitus and autoimmune thyroiditis further strengthens the relevance of this association [5].

There is a general conviction that patients with systemic rheumatic ADs (in particular with three of the main disorders such as systemic lupus erythematosus (SLE), primary Sjögren’s syndrome (pSS) and systemic sclerosis (SSc)) also have increased prevalence of CD. However, the evidence and the strength of this association is not so clear [6]. Besides descriptive case reports and case series, this hypothesis comes from some retrospective studies that provided conflicting results, as shown in Table 1, Table 2 and Table 3. Differences in patient selection and study design, limited numbers of recruited patients, inappropriate serologic screenings for CD, lack of small bowel biopsies to confirm the diagnosis and comparison of the results with an inadequate control population in the majority of these studies impaired data interpretation. 

An apparent three-fold increased risk of SLE in individuals with CD in comparison to the general population has been demonstrated in a large population-based study [6]. Another study found that SLE patients appear to have a four-fold increased risk of CD in comparison to control subjects [7]. The results of both studies, however, have been obtained in very large populations by nationwide registers, where it was impossible to validate the diagnosis of SLE in the former and CD in the latter for each case. 

Recently, a retrospective study analyzing the prevalence of different ADs in a cohort of 255 celiac subjects showed a significantly increased risk for developing at least one AD in comparison to a non-celiac cohort, with a more than two-fold risk of Hashimoto thyroiditis [8]. Among the systemic ADs evaluated, however, only a trend toward a higher prevalence of pSS was observed, since the limited sample size and the consequently very low AD prevalence found in the celiac cohort did not allow for clear conclusions to be drawn. 

Thus, we thought to verify the actual association between systemic CD and three systemic ADs with an alternative study design. In fact, we analyzed the prevalence of previously diagnosed CD in a multicenter study recruiting a large cohort of patients with well-documented diagnosis of SLE, pSS and SSc formulated in nine Italian rheumatology centers. The results were compared to those obtained in a large group of age-and sex-matched unselected adult subjects. In addition, undiagnosed CD was evaluated in the whole patient cohort, adopting a standardized protocol to accurately verify the possible presence of non-overt disease. The results of small bowel biopsies were carefully verified to confirm CD diagnosis in patients with both already diagnosed and subclinical disease [9,10].

## 2. Experimental Section

### 2.1. Study Population

The study population consisted of 1458 unselected consecutive patients fulfilling recognized classification criteria for SLE (580 patients), pSS (354 patients) and SSc (524 patients) [11,12,13] and followed up in nine Italian rheumatology centers. No patient had an overlap syndrome between the three ADs. Patients with SSc were also classified according to the limited and diffuse cutaneous subset of the disease [14]. The limited subset of SSc included both patients with early SSc (Raynaud phenomenon associated with SSc-type nailfold capillary pattern, such as megacapillaries ± avascular areas, and/or SSc selective auto-Abs) and patients with defined limited cutaneous SSc (early SSc plus cutaneous involvement distal to the elbows, knees, and clavicles) [14,15]. The results were compared with a control population represented by unselected adult sex-matched subjects, with an age ranging from 15 to 90 years, followed-up by 10 Italian primary care physicians and geographically representative to the cases. 

Among the patient cohort, cases with a previous diagnosis of CD, according to recognized diagnostic criteria [10] and checked for positive small bowel biopsy, were identified and disease-specific clinical manifestations were collected and registered in a predefined form, including a number of items described in the first column of Supplementary Table S1. Moreover, clinical and serological features specific for each connective tissue disease were systematically collected for each patient according to defined criteria, as previously described [16,17,18]. Finally, in all patient cohorts, particular attention was paid to the age at diagnosis and symptom onset of both CD and AD. The study was approved by the local ethics committee of the coordinator center (Aziende Sanitarie Umbria–CEAS (Comitato Etico Aziende Sanitarie Umbria)—study registration number 2050/12) and by the ethics committees of each participating center. Written informed consent was obtained from each patient.

### 2.2. Laboratory Methods

Serum samples from each enrolled patient were collected, aliquoted and kept frozen at −20 °C Celsius in each center. All collected sera were shipped to the coordinator center (Perugia) and tested for immunoglobulin (Ig) A anti-tissue transglutaminase (tTG) Abs, assayed with an ELISA method in which microtiter plate wells were coated with recombinant human tTG (Eu-tTG kit; Eurospital, Trieste, Italy). According to the manufacturer’s instructions, serum was diluted 1:100 and the absorbance measured at 405 nm. The cut-off value was set at 9 AU/mL. Values between 9–16 AU/mL were considered borderline and values ≥ 16 AU/mL were considered positive. Measurements were done in the same laboratory and by a single operator. Sera were thawed only once before determinations. Only sera from patients with a previously documented deficit of IgA were tested for IgG anti-deamidated synthetic gliadin peptides (DGP), according to the manufacturer’s instructions (α-GliaPep IgG, Eurospital S.p.A., Trieste, Italy). 

### 2.3. Study Protocol to Confirm CD Diagnosis

All patients without previous CD diagnosis who did not follow a gluten-free diet were screened for possible undiagnosed CD. The final CD diagnosis was performed according to validated guidelines for CD diagnosis, employing screening serological tests with very high sensitivity and specificity for CD diagnosis [10]. In detail, samples with an IgA tTG value above the cut-off (borderline and positive sera) were referred to the center of origin and locally tested again for IgA tTG and for IgA and IgG anti-endomysial Abs (EMA). Patients with confirmed high titer positivity for IgA tTG Abs, independently of IgG/IgA EMA positivity, and patients with borderline positivity for IgA tTG Abs, but IgG/IgA EMA positivity, were asked to undergo a small bowel biopsy to confirm the diagnosis of CD. At least three biopsy samples were obtained from descendent duodenum at different levels distal to the papilla. Morphological and quantitative assessments (intraepithelial lymphocyte density) were performed by experienced pathologists from each center. Morphology was categorized according to the modified Marsh classification [30].

### 2.4. Statistical Analysis

Mann–Whitney test was used to compare non-normally distributed continuous variables. Deviations from Gaussian distribution were checked using the Shapiro–Wilk test. Categorical data were examined using the χ^2^ test with Yate’s continuity correction or Fisher’s exact test. Statistical analyses were performed using IBM-SPSS® version 23.0 (IBM Corp., Armonk, NY, USA, 2015) and a two-sided *p*-value ≤0.05 was considered significant.

## 3. Results

Table 4 shows the epidemiological features of the whole population of enrolled AD patients. The control population included 14,298 subjects with an age range (15–90 years) comparable to that of the total AD population (mean ± standard deviation (SD): 53 ± 22 in controls, 53 ± 15 in AD patients, NS) and with similar sex distribution (91% females in both groups). 

Although CD prevalence was higher in each AD compared to the controls, a statistical significance was reached only for pSS patients (6.78% vs 0.64%, *p* < 0.0001), whereas only a not statistically significant trend towards increased CD prevalence was observed in SLE (1.38%, *p* = 0.058) and SSc (1.34%, *p* = 0.096) patients. However, the analysis of SSc patients according to the two recognized subtypes (limited and the diffuse cutaneous subsets (Table 5)) allowed us to observe a higher prevalence of CD among subjects with the diffuse, but not limited, type of SSc with respect to the controls (4.5% vs 0.64, *p* = 0.002). In detail, among the seven SSc patients with CD, six were characterized by the diffuse cutaneous form, four of which had circulating anti-topoisomerase I Abs, while the other one had an early SSc characterized by Raynaud phenomenon, early scleroderma pattern at nailfold videocapillaroscopy and positive antinuclear Abs at immunofluorescence, but negative anti-extractable nuclear antigen Abs. In contrast, and in agreement with this observation, none of the 209 patients with limited SSc and evidence of circulating anti-centromere Abs had had a diagnosis of CD in the past. 

The systematic evaluation of undiagnosed CD among all AD patients allowed for the detection of three subjects without a previous diagnosis of CD who were positive for IgA anti-tTG at high concentrations at screening; in particular two females with SLE (69.4 AU/mL and 190.8 AU/mL, respectively) and one female with pSS (111.5 AU/mL). High titer IgA tTG Abs were then confirmed in these three subjects when tested again at the referring center with associated positivity also for IgA EMA. No patients with borderline positivity for IgA tTG Abs had IgG/IgA EMA positivity. On the basis of our protocol, therefore, only the three patients with high titer IgA tTG underwent endoscopic examination with biopsy that showed a histological pattern diagnostic for CD according to the Marsh classification. A patient with SLE and another with pSS with positive IgA tTG Abs at screening (55 AU/mL and 17.5 AU/mL, respectively) were IgA tTG positive and EMA negative at the new determination performed in the respective center. Endoscopic examination with multiple biopsies were negative according to the Marsh classification at histopathology for both. A patient with SSc with positive IgA tTG Abs at screening (titer 17.8 U/mL) died before endoscopic examination. In addition, IgA tTG and IgA/IgG EMA were tested again in 28 patients with borderline IgA tTG positivity in each center. All patients were negative for these Abs and, according to the study protocol, biopsies were not performed. Finally, nine patients (one with SLE, four with pSS and four with SSc) presented with a deficit of IgA. Among these, the SLE patient and two out of four pSS patients had manifested CD, while the other two pSS patients and the four SSc subjects were negative for DGP Abs. 

Thus, according to these data, the overall prevalence of CD, including both subclinical and previously diagnosed CD, was 1.72% in patients with SLE, 7.06% in patients with pSS and 1.34% in patients with SSc (Table 4). All but one of the AD patients with CD were women. There was no statistically significant difference in mean age among the three groups of CD patients according to AD and CD diagnosis.

**Figure 1:**
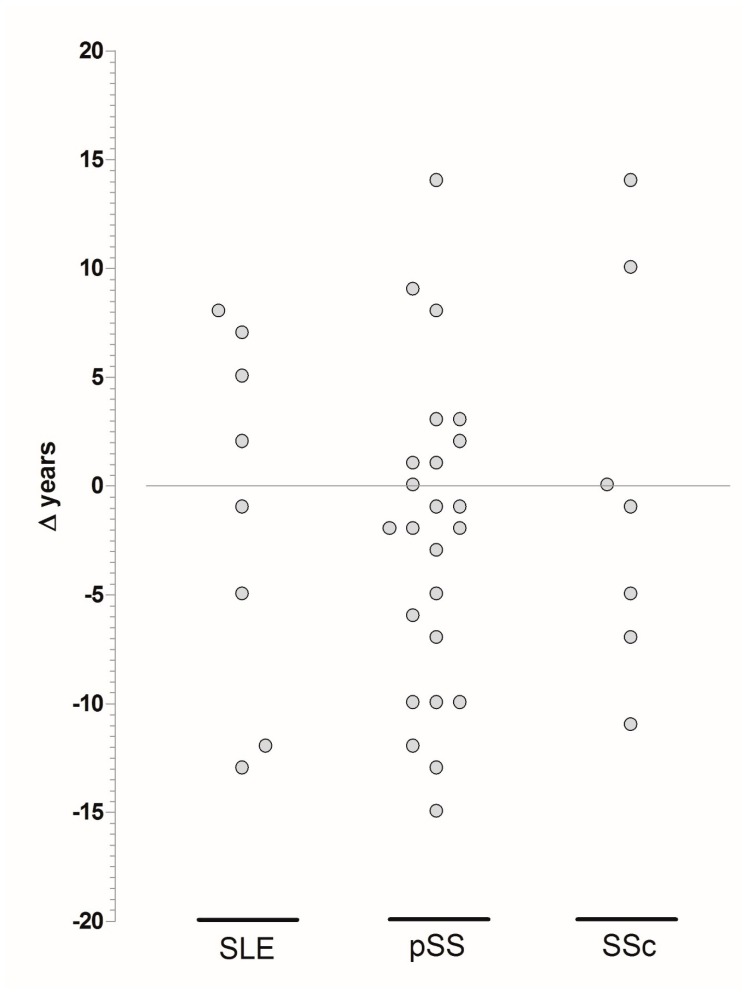
Age at CD diagnosis with respect to age at AD diagnosis.

As shown in Figure 1, the diagnosis of CD more frequently preceded that of AD, although in many patients (more than 40%), the gap between the two diagnoses was rather short (± 3 years). Notably, the age at CD diagnosis of the patients already diagnosed with AD was significantly higher than that of subjects in which the diagnosis of CD preceded that of AD (*p* = 0.03). In addition, the observation that SSc and pSS patients with CD were younger and had a lower age at AD diagnosis in comparison to patients without CD (Table 6) was of interest.

Abdominal distention was the most frequently reported symptom in the whole cohort of patients with CD, followed by chronic diarrhea and loss of appetite. Iron deficiency anemia and autoimmune thyroiditis characterized the CD phenotype in nearly half of patients, independently of AD diagnosis. The comparison of CD manifestation frequency in the three groups of patients revealed a statistically significant higher frequency of dermatitis herpetiformis in SSc patients (*p* = 0.05) (Supplementary Table S1).

The comparison of AD-specific clinical and serological features between patients with and without CD in SLE, pSS and SSc is shown in Supplementary Tables S2–S4, respectively. Many SLE patients with CD displayed a wider auto-Ab repertoire compared to SLE subjects without CD. Interestingly, the prevalence of autoimmune thyroiditis in pSS patients with CD was higher than in pSS without CD. Finally, a higher prevalence of myositis characterized SSc subjects with CD with respect to the SSc patient cohort without CD.

## 4. Discussion

The analysis of CD prevalence in each of the three ADs considered, SLE, pSS and SSc, demonstrated a higher proportion of previously diagnosed CD with respect to controls only in pSS patients with a very high prevalence (6.8%), whereas the prevalence of overt CD in SLE and SSc subjects, although approximately double with respect to controls, did not reach statistical significance. Regarding SSc, however, the evidence that nearly all celiac SSc patients in our cohort were characterized by a definite diffuse cutaneous form of the disease, whereas anti-centromere Abs, a marker of the limited cutaneous scleroderma subset, were never detected in these subjects. The higher prevalence of myositis found in CD SSc patients appears to be in agreement with this finding. Similar results have been described in a small cohort of Italian SSc patients with CD, of which 80% had the diffuse cutaneous type of the disease [27]. Another study showed that IgA-EMA Abs were negative in 105 SSc patients, the majority of which had the limited form of the disease [28]. Our observation, highlighting a very high prevalence of CD among subjects with diffuse SSc, may open a number of intriguing questions from both pathogenic and clinical points of view, since it may suggest different immunological pathways underlying SSc subtypes and possible shared mechanisms between CD and the diffuse, but not limited, form of scleroderma. In this setting, the higher prevalence of dermatitis herpetiformis, considered “the CD of the skin”, in CD SSc patients may represent an additional intriguing finding that may be supported by potential common autoimmune mechanisms underlying the three disorders [31]. 

The evaluation of subclinical CD in AD patients allowed us to find three additional CD patients (two with SLE and one with pSS) with a consequent overall CD prevalence of 2.9% in the total AD patient group. This is clearly higher than that reported in a consistent population of Italian adult subjects, in which the overall prevalence of CD, identified by a mass screening project, was 0.7% [32]. In this setting, however, it is interesting to note that our investigation showed a very high ratio between the proportion of previously diagnosed and overall CD prevalence (93%) which appears to be substantially different from that described some years ago in general populations recruited from different European countries, ranging from 6–24% [32]. Although the low number of subclinical CD found in AD patients may be related to a bias due to the particular selection of our population, it could mirror increased awareness of CD in recent years. We are aware that a limitation of this study is represented by the fact that a systematic screening for subclinical CD in the control group, a source of invaluable information from a general medical point of view, was not feasible in this study for a number of technical reasons. However, we believe that the lack of this data does not invalidate the absence of a statistically significant difference in CD prevalence between controls and SLE or all SSc patients, and it does not appear relevant in influencing the statistical significance of the high CD prevalence found in pSS and diffuse cutaneous SSc compared to controls, due to the big differences between these groups. 

Little is known about the possible immunopathogenic basis of CD and AD association [4]. Undoubtedly, a common genetic background may play a key role in favoring CD association with a number of ADs, in particular with pSS [33]. Intriguingly, autoimmune thyroiditis, the most frequent organ-specific AD associated with CD, was more prevalent in our pSS patients with CD than those without CD. Another important pathogenic contribution to this association may be given by alteration of the microbiota composition or dysbiosis which may contribute to inducing and modulating systemic inflammation in both CD and ADs [34], and in particular in pSS and SSc, as recently suggested [35,36].

In this setting, it is conceivable to postulate that, in both CD and pSS, environmental factors [37,38] may promote abnormal immune responses, mononuclear cell infiltration and organ tissue damage of the small bowel in CD, exocrine glands in pSS and possibly the thyroid in Hashimoto disease. Both disorders are characterized by aberrant auto-Ab production and antinuclear Abs (ANA) have been found to be increased in patients with CD and non-celiac wheat sensitivity [39,40,41]. This fits with the presence of a wide variety of auto-Abs found in SLE subjects with CD (Supplementary Table S4) and the high frequency of low titer and unspecific IgA Abs against tTG found in our series; these findings fit with previous studies demonstrating frequent false positive serology for anti-gliadin, tTG Abs and EMA in patients with systemic ADs (Table 1, Table 2 and Table 3). It is of interest to note that non-celiac patients were characterized by tTG Abs positivity and EMA negativity. In this setting, EMA determination may be performed in patients with systemic ADs and suspected CD while considering the need for duodenal biopsy. 

We are aware that the age at CD or AD diagnosis may not correspond to the actual disease onset and we cannot draw definitive conclusions about the relationship between the two disorders or the consequent pathogenic implications. However, in agreement with the results of other studies, CD diagnosis usually preceded the diagnosis of AD in the majority of our patients, thereby suggesting CD as predisposing factor for AD development. Moreover, in agreement with a previously published study [26], we found that the age of both pSS and SSc patients with CD was lower than that of the correspondent patient group without CD, thereby supporting the idea that CD may be able to promote and accelerate AD development. In this regard, the demonstration that the risk to develop ADs in CD subjects is increased in patients diagnosed with CD in childhood or at young age [42] is of interest. It is noteworthy, however, that the time gap between the diagnosis of the two disorders was rather short in a number of our patients. In addition, we also found that patients with CD diagnosis subsequent to that of AD were older than subjects with an antecedent CD diagnosis. These observations may also suggest that AD manifestations may mask CD symptoms with a consequent delay in CD diagnosis.

In fact, it is important to consider that CD association with systemic ADs is relevant either because of a detrimental effect on the clinical burden of CD (and vice versa) or because symptoms of secondary autoimmunity can be the sole presentation of CD. In a large proportion of cases, indeed, the disease remains clinically silent, particularly in the elderly [43], and the only manifestation is associated diseases [44]. The classical example is represented by CD diagnosis upon screening after diagnosis of type I diabetes mellitus or thyroiditis. Thus, the diagnosis of CD may represent a major clinical challenge in patients with systemic ADs due to the high variability of gastrointestinal symptoms characterizing these disorders [45]. Indeed, the most frequent signs and symptoms characterizing CD at diagnosis in our series were represented by signs of gastrointestinal involvement, such as abdominal distention, chronic diarrhea and loss of appetite. In ADs, such as pSS or SSc, various gastrointestinal manifestations occur due to decreased saliva production, esophageal dysmotility, gastroparesis, chronic atrophic gastritis, impaired pancreatic function, fibrous involvement of intestinal smooth muscle with consequent loss of peristalsis or intraluminal bacterial overgrowth, thus mimicking CD symptoms. Therefore, the occurrence of these ADs may cause impaired interpretation of the etiology of gastrointestinal symptoms in subjects with subclinical CD. In addition, the frequent occurrence of iron deficiency anemia may represent another important confounding factor.

It is well known that the risk of developing CD complications, including ADs, may be closely related to the duration of exposure to gluten and reduced by rigorous adherence to a gluten-free diet [42,46]. Unfortunately, we are aware that our study does not provide information on gluten-free diet compliance in CD patients, which is key datum in order to evaluate its effect on AD. However, we believe that the recognition of signs and symptoms potentially underlying CD in patients with systemic ADs is of great importance. Awareness of the actual prevalence of CD in patients with systemic AD is important when the long-term clinical implications of an unrecognized CD in the general population is considered [47]. A nearly four-fold increased risk of all-cause mortality in subjects with unrecognized CD compared to serologically negative controls has been shown [48]. In addition, CD, similarly to pSS, may be associated with a major risk of developing non-Hodgkin lymphoma [49,50], thereby stressing not only the intriguing potential common pathogenic mechanisms, but also the importance to diagnose concomitant CD in pSS patients [49]. In this setting, analysis of intestinal biopsies of pSS patients with CD with additional specific immunologic methods will be very interesting to highlight common pathogenic pathways underlying the increased risk of CD in these patients.

## 5. Conclusions

The prevalence of CD appears to be strongly increased in patients with pSS, while increased prevalence in SSc seems to be confined to the diffuse cutaneous form of the disease. The actual association of CD with SLE, on the contrary, remains uncertain. A routine screening for CD may be recommended in patients suffering from pSS and, probably, diffuse SSc, particularly in younger patients. Besides the occurrence of common CD features, such as iron-deficiency anemia or weight loss, the coexistence of autoimmune thyroiditis in pSS subjects may represent an additional risk factor for the presence of subclinical CD. Additional studies are ongoing in order to further define and validate these data.

## Figures and Tables

**Table 1:** Celiac disease prevalence in systemic lupus erythematosus.

Author Year [ref]	Pts, n Country	Screening for CD	Ab Result (Prevalence)	Adopted Criteria to Perform SBB	SBB Positive Results (Prevalence)
Rensh M 2001 [19]	103 USA	IgA/IgG AGA and EMA	24 pts AGA^+^ (all pts EMA^−^)(23%)	AGA^+^ and/or EMA^+^	0/24 (0%)
Luft LM 2003 [20]	50 USA	IgA tTG IgA EMA in tTG^+^	3 pts tTG^+^/EMA^–^ (6%)	Retrospective data	ND
Marai I 2004 [21]	100 Italy/ Israel	IgA/IgG tTG IgA/IgG EMA in tTG^+^ or HLA-DQ2/8 in EMA^–^	3 pts tTG^+^ (3%) (1 EMA^+^ and 2 EMA^–^/DQ2/8^–^)	tTG^+^ and EMA^+^ or EMA^–^/DQ2/8^+^	1/1 (1%)
Koehne V 2010 [22]	69 Brazil	IgA/IgG AGA and IgA EMA IgA-tTG in EMA^+^	2 pts AGA^+^/EMA^–^ (3%) 3 pts EMA^+^/tTG^–^ (4%)	AGA^+^ or EMA^+^	0/5 (0%)
Ben Abdelghani K 2012 [23]	24 Tunisia	AGA and tTG	5 pts AGA^+^/tTG^–^ (21%) 2 pts AGA^+^/tTG^+^ (8%)	All pts	1/24 (AGA^+^/tTG^+^) (4%)

AGA, anti-gliadin Abs; EMA, anti-endomysial Abs; tTG, tissue transglutaminase Abs; ND, not done; Pts, patients; SBB, small bowel biopsy; IgA: Immunoglobulin A; IgG: Immunoglobulin G.

**Table 2:** Celiac disease prevalence in Sjögren’s syndrome.

Author Year [ref]	Pts, n Country	Screening for CD	Ab Results (Prevalence)	Adopted Criteria to Perform SBB	SBB Positive Results (Prevalence)
Iltanen S 1999 [24]	34 Finland	ND	3 pts EMA^+^ (9%) 13 pts AGA^+^ (38%) 19 pts DQ2^+^ (56%)	All pts	5 (15%)
Bizzaro N 2003 [25]	100 Italy/ Israel	IgA/IgG tTG IgA/IgG EMA in tTG^+^ or HLA-DQ2/8 in EMA^−^	1 pt tTG^+^/ EMA^−^/DQ2/8^−^ (1%)	tTG^+^ and EMA^+^ or EMA^−^/DQ2/8^+^	ND
Luft LM 2003 [20]	50 USA	IgA tTG IgA EMA in tTG^+^	5 pts tTG^+^/EMA^+^ (10%) 1 pt tTG^+^/EMA^–^ (2%)	Retrospective data	5 (tTG^+^/EMA^+^) (10%)
Szodoray P 2004 [26]	111 Hungary	IgA tTG, IgA EMA, IgG/IgA AGA	6 pts with serology^+^ (not specified) (5%)	tTG^+^ and/or EMA^+^ and/or AGA^+^	5 (4.5%)

AGA, anti-gliadin Abs; EMA, anti-endomysial Abs; tTG, tissue transglutaminase Abs; ND, not done; Pts, patients; SBB, small bowel biopsy.

**Table 3:** Celiac disease prevalence in systemic sclerosis.

Author Year [ref]	Pts, n Country	Screening for CD	Ab Results (Prevalence)	Adopted Criteria to Perform SBB	SBB Positive Results (Prevalence)
Luft LM 2003 [20]	30 USA	IgA tTG IgA EMA in tTG^+^	2 pts tTG^+^/EMA^+^ (7%)	Retrospective data	ND
Bizzaro N 2003 [25]	100 Italy/ Israel	IgA/IgG tTG IgA/IgG EMA in tTG^+^ or HLA-DQ2/8 in EMA^−^	1 pt tTG^+^/ EMA^−^/DQ2/8^–^ (1%)	tTG^+^ and EMA^+^ or EMA^−^/DQ2/8^+^	ND
Rosato E 2009 [27]	50 Italy	IgA/IgG tTG IgA/IgG EMA in tTG^+^	2 pts tTG^+^/EMA^+^ 3 pts tTG^+^/EMA^−^ (10%)	tTG^+^/EMA^+/–^	4/5 (1 pt refused) (8%)
Nisihara R 2011 [28]	105 Brazil	IgA EMA	All pts EMA^–^	NA	ND
Forbess LJ 2013 [29]	72 USA	IgA/IgG tTG and IgA/IgG DGP EMA in tTG^+^ and/or DGP^+^	1 tTG^+^/EMA^−^ 2 DGP^+^/EMA^−^ (4%)	tTG^+^ and/or DGP^+^	0/3 (1 pt died) (0%)

AGA, anti-gliadin Abs; EMA, anti-endomysial Abs; tTG, tissue transglutaminase Abs; DGP, anti-deamidated gliadin peptide Abs; ND, not done; Pts, patients; SBB, small bowel biopsy.

**Table 4:** Epidemiological features of, and celiac disease prevalence in patients with systemic autoimmune diseases and the control population.

	Normal Controls	SLE	pSS	SSc
Subjects, *n*	14,298	580	354	524
Age, mean ± SD (range)	53 ± 22 (15–90)	**46 ± 13** (19–83)	55 ± 12 (21–90)	**61 ± 14** (15–87)
Female (%)	91	89	97	90
Previously diagnosed CD, *n*	91	8	24	7
Previously diagnosed CD prevalence, % (95% CI)	0.64 (0.5–0.8)	1.38 (0.7–2.7)	**6.78** (4.6–9.9)	1.34 (0.7–2.7)
Subclinical CD, *n*	ND	2	1	0
Overall CD prevalence, % (95% CI)	NA	1.72 (0.9–3.1)	7.06 (4.8–10.2)	1.34 (0.7–2.7)
Age at AD diagnosis, mean ± SD (range)	NA	33 ± 14 (3–79)	48 ± 12 (20–77)	51 ± 14 (13–81)

Results in bold are statistically different from controls. ND, not done; NA, not applicable; CD, celiac disease; AD, autoimmune disease; SD, standard deviation; CI: Confidence interval.

**Table 5:** Epidemiological features of, and celiac disease prevalence in patients with systemic sclerosis according to different clinical subsets and specific serology.

SSc Subset	Diffuse					Limited				
Auto-Abs	All	ATA+ *	ACA+ §	Other Auto-Abs+ ^	None	All	ATA+	ACA+	Other Auto-Abs+	None
Subjects, n	134	94	8	26	21	390	**77**	209	90	51
% with respect to all subjects	-	70.1	5.9	19.4	19.4	-	19.7	53.6	23.1	13.1
Age, mean ± SD	57 ± 14	57 ± 14	63 ± 9	58 ± 15	53 ± 14	62 ± 13	60 ± 15	64 ± 12	63 ± 13	59 ± 13
Previously diagnosed CD, n	6	4	0	2	0	1	0	0	1	0
Previously diagnosed CD prevalence, % (95% CI)	**4.5** **(2.1–9.4)**	**4.3** **(1.7–10.4)**	0	**7.7** **(2.1–24.1)**	0	0.3 (0.1–1.4)	0	0	1.1 (0.5–1.9)	0

Results in bold are statistically different from controls. * Anti-topoisomerase I Abs. § Anti-centromere Abs. ^ Other SSc-related auto-Abs and/or antinuclear Abs detected at indirect immunofluorescence test. SSc, systemic sclerosis; CD, celiac disease; auto-Abs, autoantibodies; ATA, anti-topoisomerase antibody; ACA, anti-centromere antibody.

**Table 6:** Age at diagnosis and age at symptom onset of SLE, pSS and SSc patients with CD in comparison to those without evidence of CD.

Disease	Age, Mean ± SD (Range)	Age at Diagnosis, Mean ± SD (Range)	Age at Symptom Onset, Mean ± SD (Range)
SLE–CD (*n* = 10)	39 ± 8 (25–50)	31 ± 11 (14–47)	26 ± 12 (14–47)
SLE–non-CD (*n* = 570)	46 ± 13 (19–83)	33 ± 14 (3–79)	31 ± 14 (2–72)
pSS–CD	**49 ± 9 (34–76)**	**43 ± 10 (27–73)**	40 ± 10 (27–74)
pSS–non-CD	55 ± 12 (21–90) *	**48 ± 12 (20–77) ^**	44 ± 12 (18–76)
SSc–CD	**46 ± 16 (15–63)**	**40 ± 14 (13–61)**	41 ± 13 (28–60)
SSc–non-CD	61 ± 13 (22–87) *	**51 ± 14 (0–81) ^**	46 ± 15 (0–81)

Statistically significant differences in bold (* *p* ≤ 0.01, ^ *p* ≤ 0.04, CD vs non-CD).

## Supplementary Materials

The following are available online at https://www.mdpi.com/2077-0383/8/4/540/s1. Table S1: Frequency of celiac disease-specific manifestations in the three groups of AD patients, Table S2: Frequency of clinical and laboratory features in SLE patients with CD in comparison to those without evidence of CD, Table S3: Frequency of clinical and laboratory features in pSS patients with CD in comparison to those without evidence of CD, Table S4: Frequency of clinical and laboratory features in SSc patients with CD in comparison to those without evidence of CD.
