# Supplementary material for: Celiac Disease Prevalence Is Increased in Primary Sjögren’s Syndrome and Diffuse Systemic Sclerosis: Lessons from a Large Multi-Center Study
Source: J Clin Med. 2019 Apr 19;8(4):540. doi: 10.3390/jcm8040540 (PMC6517955; doi:10.3390/jcm8040540)
Supplement: Supplementary file 1 [file jcm-08-00540-s001.pdf]

Journal of
Clinical Medicine

MDPI

Supplementary Table 1. Frequency of celiac disease-specific manifestations in the three groups of AD patients.

CD features, *n* (%)	Total (*n* = 42)	SLE (*n* = 10)	pSS (*n* = 25)	SSc (*n* = 7)	*p* value
Abdominal distention	22 (52%)	6 (60%)	13 (52%)	3 (43%)	0.8
Iron deficiency anemia	19 (45%)	5 (50%)	10 (40%)	4 (57%)	0.7
Autoimmune thyroiditis	19 (45%)	4 (40%)	12 (48%)	3 (43%)	0.9
Chronic diarrhea	15 (36%)	4 (40%)	9 (36%)	2 (29%)	0.9
Osteopenia	12 (29%)	2 (20%)	8 (32%)	2 (29%)	0.8
Loss of appetite	11 (26%)	5 (50%)	4 (16%)	2 (29%)	0.1
Constipation	8 (19%)	1 (10%)	6 (24%)	1 (14%)	0.6
Osteoporosis	6 (14%)	1 (10%)	5 (20%)	0 (0%)	0.4
Steatorrhea	5 (12%)	0 (0%)	3 (12%)	2 (29%)	0.2
Muscle hypotrophy	5 (12%)	2 (20%)	2 (8%)	1 (14%)	0.6
Depression	5 (12%)	2 (20%)	1 (4%)	2 (29%)	0.1
First-degree family members	4 (9%)	1 (10%)	2 (8%)	1 (14%)	0.7
Dermatitis herpetiformis	3 (7%)	0 (0%)	1 (4%)	2 (29%)	**0.05**
Peripheral neuropathy	3 (7%)	0 (0%)	2 (8%)	1 (14%)	0.5
Infertility/recurrent miscarriages	3 (7%)	2 (20%)	0 (0%)	1 (14%)	0.08
Atrophic glossitis	3 (7%)	1 (10%)	2 (8%)	0 (0%)	0.7
Epilepsy	1 (2%)	1 (10%)	0 (0%)	0 (0%)	0.2
Ataxia	0 (0%)	0 (0%)	0 (0%)	0 (0%)	NA
Autoimmune hepatitis	0 (0%)	0 (0%)	0 (0%)	0 (0%)	NA
Primary biliary cirrhosis	0 (0%)	0 (0%)	0 (0%)	0 (0%)	NA

Statistically significant differences in bold.

NA, not applicable; CD, celiac disease; AD, autoimmune disease; SLE, systemic lupus erythematosus; pSS, primary Sjögren syndrome; SSc, systemic sclerosis.

Supplementary Table 2. Frequency of clinical and laboratory features in SLE patients with CD in comparison to those without evidence of CD.

SLE features	SLE–CD (*n* = 10)	SLE–non-CD (*n* = 570)	*p* value
Arthritis/arthralgia, *n* (%)	6 (60%)	438 (77%)	0.2
Malar rash, *n* (%)	3 (30%)	261 (46%)	0.3
Photosensitivity, *n* (%)	3 (30%)	280 (49%)	0.3
Discoid lupus erythematosus, *n* (%)	0 (0%)	30 (5%)	1.0
Oral ulcers, *n* (%)	0 (0%)	91 (16%)	0.4
Raynaud phenomenon, *n* (%)	2 (20%)	187 (33%)	0.5
Serositis, *n* (%)	4 (40%)	114 (20%)	0.1
Lung involvement, *n* (%)	0 (0%)	36 (6%)	1.0
Pulmonary arterial hypertension, *n* (%)	1 (10%)	8 (1%)	0.1
Lupus glomerulonephritis, *n* (%)	3 (30%)	217 (38%)	0.7
Myositis, *n* (%)	1 (10%)	14 (2%)	0.2
Peripheral nervous system involvement, *n* (%)	1 (10%)	39 (7%)	0.5
Psychosis, *n* (%)	0 (0%)	11 (2%)	1.0
Epilepsy, *n* (%)	1 (10%)	21 (4%)	0.3
Leukopenia, *n* (%)	5 (50%)	174 (30%)	0.3
Immune-hemolytic anemia, *n* (%)	3 (30%)	76 (13%)	0.1
Thrombocytopenia, *n* (%)	3 (30%)	97 (17%)	0.4
Low complement, *n* (%)	6 (60%)	320 (56%)	1.0
IgM/IgG anti-cardiolipin, *n* (%)	3 (30%)	129 (23%)	0.7
IgM/IgG anti-β2 glycoprotein I, *n* (%)	2 (20%)	94 (16%)	0.7
Lupus anticoagulant, *n* (%)	0 (0%)	90 (16%)	0.4
Anti-Sm, *n* (%)	3 (30%)	82 (14%)	0.2
Anti-RNP, *n* (%)	1 (10%)	95 (17%)	1.0
Anti-dsDNA, *n* (%)	0 (0%)	1 (0.2%)	0.6
Anti-Ro52/Ro60, *n* (%)	1 (10%)	190 (33%)	0.2
Other autoantibodies, *n* (%)	**4 (40%)**	**30 (5%)**	**0.002**

Statistically significant differences in bold.

CD, celiac disease; SLE, systemic lupus erythematosus.

Supplementary Table 3. Frequency of clinical and laboratory features in pSS patients with CD in comparison to those without evidence of CD.

pSS features	pSS–CD (n = 25)	pSS–non-CD (n = 329)	p value
Xerostomia, *n* (%)	21 (84%)	286 (87%)	0.5
Xerophtalmia, *n* (%)	24 (96%)	302 (92%)	0.7
Parotid swelling, *n* (%)	7 (28%)	132 (40%)	0.3
Vasculitic purpura, *n* (%)	1 (4%)	37 (11%)	0.5
Arthritis/arthralgia, *n* (%)	16 (64%)	210 (64%)	1.0
Raynaud phenomenon, *n* (%)	9 (36%)	82 (25%)	0.2
Serositis, *n* (%)	0 (0%)	20 (6%)	0.4
Lung involvement, *n* (%)	1 (4%)	23 (7%)	1.0
Kidney involvement, *n* (%)	1 (4%)	12 (4%)	1.0
MGUS, *n* (%)	1 (4%)	16 (5%)	1.0
Lymphoma, *n* (%)	1 (4%)	11 (3%)	0.6
Myositis, *n* (%)	0 (0%)	1 (0.3%)	1.0
Central nervous system involvement, *n* (%)	0 (0%)	12 (4%)	1.0
Peripheral nervous system involvement, *n* (%)	2 (8%)	50 (15%)	0.5
Autoimmune thyroiditis, *n* (%)	**12 (48%)**	**81 (25%)**	**0.02**
Autoimmune hepatitis, *n* (%)	0 (0%)	9 (3%)	1.0
Low complement, *n* (%)	4 (16%)	55 (17%)	1.0
Leukopenia, *n* (%)	8 (32%)	78 (24%)	0.3
Hypergammaglobulinemia, *n* (%)	9 (37%)	133 (40%)	0.8
Cryoglobulins, *n* (%)	1 (4%)	9 (3%)	0.5
IgM/IgG anti-cardiolipin, *n* (%)	1 (4%)	12 (4%)	1.0
Anti-Ro52/Ro60, *n* (%)	21 (84%)	244 (74%)	0.3
Anti-La, n (%)	10 (40%)	141 (43%)	0.8

Statistically significant differences in bold.

MGUS, monoclonal gammopathy of undetermined significance; CD, celiac disease; pSS, primary Sjögren syndrome.

Supplementary Table 4. Frequency of clinical and laboratory features in SSc patients with CD in comparison to those without evidence of CD.

SSc features	SSc–CD<br>(*n* = 7)	SSc–non-CD<br>(*n* = 517)	*p* value
Raynaud phenomenon, *n* (%)	7 (100%)	467 (90%)	1.0
Arthritis/arthralgia, *n* (%)	2 (29%)	219 (42%)	0.7
Tendon friction, *n* (%)	2 (29%)	63 (12%)	0.2
Skin ulcers, *n* (%)	2 (29%)	185 (36%)	1.0
Serositis, *n* (%)	1 (14%)	29 (6%)	0.3
Pulmonary fibrosis, *n* (%)	2 (29%)	201 (39%)	0.7
Pulmonary arterial hypertension, *n* (%)	1 (14%)	106 (20%)	1.0
Scleroderma renal crisis, *n* (%)	1 (14%)	7 (1%)	0.1
Gastrointestinal involvement, *n* (%)	3 (43%)	255 (49%)	1.0
Myositis, *n* (%)	**2 (29%)**	**18 (3%)**	**0.03**

Statistically significant differences in bold.

CD, celiac disease; SSc, systemic sclerosis.
